# Ressonância Magnética Cardíaca para Avaliar a Eliminação Completa do Substrato após a Ablação Endocárdica da Taquicardia Ventricular na Doença de Chagas

**DOI:** 10.36660/abc.20230421

**Published:** 2024-02-16

**Authors:** Mauricio I. Scanavacca, Rodrigo M. Kulchetscki, Carlos E. Rochitte, Cristiano F. Pisani

**Affiliations:** 1 Instituto do Coração do Hospital das Clínicas Faculdade de Medicina Universidade de São Paulo São Paulo SP Brasil Instituto do Coração do Hospital das Clínicas da Faculdade de Medicina da Universidade de São Paulo - Departamento de Arritmia, São Paulo , SP – Brasil; 2 Instituto do Coração do Hospital das Clínicas Faculdade de Medicina Universidade de São Paulo São Paulo SP Brasil Instituto do Coração do Hospital das Clínicas da Faculdade de Medicina da Universidade de São Paulo - Departamento de Imagem Cardiovascular, São Paulo , SP – Brasil

**Keywords:** Doença de Chagas, Taquicardia Ventricular, Espectroscopia de Ressonância Magnética, Técnicas de Ablação/métodos

## Introdução

O desfecho principal da ablação da taquicardia ventricular (TV) inclui a eliminação do substrato anormal no remapeamento e promover a não indução da TV pela estimulação ventricular programada (EVP). ^1 , 2^

A ressonância magnética cardíaca (RMC) tem sido crescentemente utilizada como uma ferramenta para localizar o substrato arritmogênico dos pacientes com TV relacionada a cicatriz. As imagens podem ser integradas com mapeamento eletroanatômico para direcionar a ablação aos canais responsáveis pelos circuitos de reentrada, otimizando os resultados da ablação. ^3^ No entanto, até o momento, a RMC não foi usada para confirmar a eliminação completa do substrato nem a eficácia da ablação da TV.

Neste artigo, relatamos um caso de um paciente com cardiomiopatia chagásica ^4 , 5^ que foi submetido à ablação endocárdica da TV, guiada por mapeamento da TV e modificação do substrato. Descrevemos, pela primeira vez, o uso da RMC após o procedimento para confirmação da eliminação do substrato da TV.

## Apresentação do Caso

**Figura 1:**
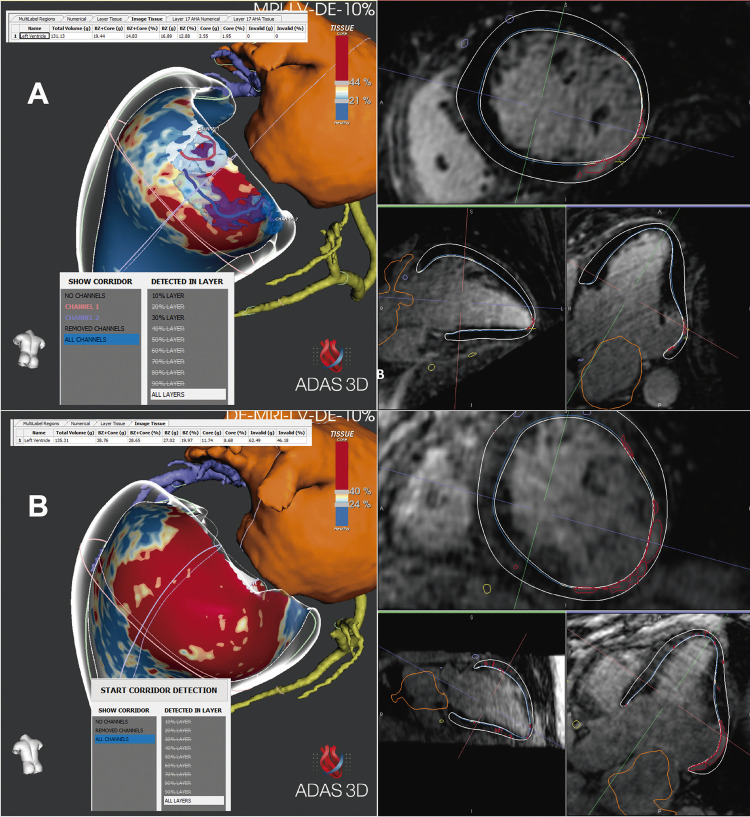
– A) Ressonância magnética cardíaca tridimensional com realce tardio processada no programa ADAS ® , mostrando uma cicatriz heterogênea na região ínfero-basal, com um centro e uma zona periférica, volume da cicatriz de 19,4g (14,83% da massa do ventrículo esquerdo), e dois corredores, o corredor #1, próximo ao anel mitral, e o corredor #2, na lateral da cicatriz. B) mesma reconstrução, mas após a ablação; observe a extensão lateral da cicatriz, relacionada à área da ablação, com massa da cicatriz de 38,8g (26,6% da massa do ventrículo esquerdo), e ausência de corredores

Paciente de 68 anos, do sexo masculino, com doença de Chagas, apresentou com palpitações causadas por TV monomórfica sustentada (Figura 1A). A TV foi interrompida por cardioversão elétrica. O paciente relatou um episódio anterior de síncope e recebia amiodarona (200mg) pela presença de TV não sustentada detectada no Holter. O ecocardiograma mostrou Fração de Ejeção Ventricular Esquerda (FEVE) de 40%, dimensões do ventrículo esquerdo de 67 x 58 mm, acinesia da parede lateral e hipocinesia da parede inferior. O conjunto dos achados sugere que o paciente estava no estágio B2 da doença, de acordo com a classificação da insuficiência cardíaca na doença de Chagas. ^5^ Uma tomografia computadorizada (TC) abdominal foi realizada para avaliação de megacólon. Uma RMC tridimensional com Realce Tardio (RMC-3D-RT) foi realizada (Phillips ^®^ Achieva 1.5T system), usando a ferramenta *Navigator* para compensação respiratória (sequência 3D-LGE), e os arquivos crus foram exportados para o programa ADAS ^®^ (Galgo Medical, Barcelona, Espanha) para processamento da imagem para avaliar a distribuição da cicatriz e presença de corredores. Foram identificados uma cicatriz inferior, lateral, basal e apical, com 19,4g (14,83% da massa do ventrículo esquerdo) na área da margem e centro, e dois corredores relacionadas à cicatriz inferior e basal (Figura 1A). A massa do corredor foi 1,72g e ambos se encontravam na camada endocárdica (10 e 30%), em que o corredor número 1 estava na porção lateral da cicatriz, e o número 2 relacionava-se ao anel mitral.

**Figura 2:**
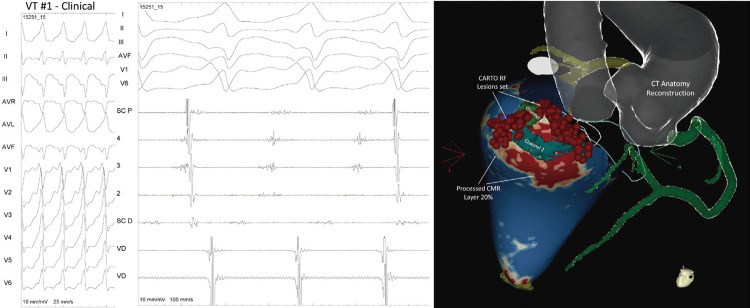
– Taquicardia ventricular clínica induzida no procedimento, mostrando ativação do seio coronariano e mapa de ativação CARTO ® sugestivo de um circuito no istmo mitral; o mapeamento de voltagem mostrou cicatriz na região ínfero-basal, com potenciais fragmentados na lateral da cicatriz e próximo ao anel mitral. Existiam três diferentes morfologias de taquicardia ventricular induzida durante o procedimento. Imagens de ressonância magnética cardíaca tridimensional com realce tardio (camada de 20%) e anatomia por tomografia computadorizada foram exportadas offline para CARTO®, após processamento pelo ADAS®, mostrando que o conjunto de lesões por radiofrequência estava relacionado à cicatriz heterogênea e presença de corredores

O paciente foi encaminhado para ablação da TV usando o sistema CARTO 3 ^®^ (Biosense Webster/Johnson & Johnson, EUA) e recrutado para um ensaio clínico, ^6^ em que foi alocado aleatoriamente para o grupo “somente ablação endocárdica”. O paciente foi submetido ao mapeamento do endocárdio, que mostrou cicatriz inferior, lateral, e basal com potenciais fragmentados na porção lateral da cicatriz e próxima ao seio coronário. A TV clínica, hemodinamicamente estável, foi induzida (S1 600ms S4 310ms), com atividade diastólica sugestiva de uma TV do istmo mitral, também correlacionada ao corredor número 2. A ablação endocárdica (30W, tempo RF total: 24m4s) foi realizada, com interrupção da TV; a ablação foi estendida, eliminando todos os sinais fragmentados próximo ao anel ( Figura 2 ). Após o remapeamento, potenciais anormais ainda eram observados na parte lateral da cicatriz na região do corredor número 1. Assim, ablação adicional foi realizada nessa porção, alcançando completa homogeneização do substrato. Uma EVP realizada após a ablação com S1 600ms e 430ms até S4 não induziu nenhuma TV. O paciente recebeu alta sem Cardioversor Desfibrilador Implantável (CDI) e agendado para reavaliação em um mês.

Um mês depois, uma segunda RMC-3D-RT foi realizada, com o mesmo equipamento usado anteriormente, que agora mostrou 38,8g (28,6% da massa do VE) de cicatriz. Um ponto importante é que não foram detectados canais no interior da cicatriz ( Figura 1 B). Durante um segundo estudo eletrofisiológico usando o mesmo protocolo de EVP, ainda não foi possível a indução de TV. O paciente recebeu alta sem implante de CDI, mantendo o uso de amiodarona 200mg. Em um seguimento de cinco anos, o paciente foi mantido sob a mesma dose de amiodarona, com FEVE de 48% no ecocardiograma, sem recorrência de TV, e sem implantação de CDI.

## Discussão

Embora o desfecho clássico da ablação da TV seja a impossibilidade de se induzir arritmias após o procedimento, ^7 , 8^ a completa eliminação de todos os eletrogramas anormais ^9^ também se correlaciona com um risco mais baixo de recorrência de TV. Apesar da possibilidade de recorrência de TV devido à progressão da doença, a eliminação incompleta do substrato arritmogênico parece ser o principal mecanismo. ^10^ Uma RMC pré-ablação é comumente usada para localizar os corredores dentro da cicatriz, um achado que se correlaciona com piores desfechos na cardiomiopatia chagásica crônica, e pode guiar a ablação da TV. ^5 , 11^ Neste artigo, descrevemos o uso da RMC repetida após a ablação para confirmar que todos os corredores foram eliminados.

Nosso paciente apresentou uma TV hemodinamicamente tolerada, depressão moderada (40%) da FEVE, sem cicatriz extensa (19g), e apenas dois corredores identificados na RMC. Durante a ablação, a TV foi mapeada e interrompida sem reindução, e todos os potenciais anormais foram eliminados. Na ocasião, decidimos não implantar um CDI, e repetir uma RMC controle em um mês, seguida de um estudo eletrofisiológico. A RMC de pós-ablação confirmou que todos os corredores foram eliminados, juntamente com um estudo eletrofisiológico negativo, permitindo a alta do paciente sem um CDI. ^12^ Cinco anos depois, o paciente continuava livre de síncope ou recorrência de TV, sem depressão da função ventricular esquerda (48%), mantendo o uso de uma dose moderada de amiodarona (200mg), a mesma que ele usava antes dos primeiros episódios de TV e sem CDI.

O tempo necessário para que as lesões de radiofrequência sejam identificadas na RMC é completamente desconhecido, uma vez que lesões agudas podem apresentar edema e regiões de obstrução microvascular. Nesse caso, nós repetimos a RMC – na mesma máquina e pela mesma equipe -, bem como o estudo eletrofisiológico um mês após o procedimento index, um período que deveria mostrar um padrão mais consolidado da lesão. ^13^

É notável que, uma vez que a doença de Chagas é uma condição progressiva, com uma história natural de fibrose progressiva, ^5 , 14^ novos circuitos de TV reentrante podem se formar, assim a estratégia de não usar CDI precisa ser testada em um ensaio clínico randomizado.

Esta carta científica, no entanto, corrobora a aplicação investigativa sobre o uso de uma RMC controle para avaliar a modificação do substrato após a ablação, permitindo que pacientes com cicatrizes menos extensas e sem disfunção grave do ventrículo esquerdo possam continuar sem CDI com segurança.
