# Supplementary material for: Ressonância Magnética Cardíaca para Avaliar a Eliminação Completa do Substrato após a Ablação Endocárdica da Taquicardia Ventricular na Doença de Chagas
Source: Arq Bras Cardiol. 2024 Feb 16;121(1):e20230421. [Article in Portuguese] doi: 10.36660/abc.20230421 (PMC11098567; doi:10.36660/abc.20230421)
Supplement: Translation

# Translation: Cardiac Magnetic Resonance to Evaluate Complete Substrate Elimination after Endocardial Ventricular Tachycardia Ablation in Chagas Disease

**Authors:** Mauricio I. Scanavacca, Rodrigo M. Kulchetscki, Carlos E. Rochitte, Cristiano F. Pisani

**Affiliations:** 1 Brazil Instituto do Coração do Hospital das Clínicas da Faculdade de Medicina da Universidade de São Paulo - Departamento de Arritmia, São Paulo, SP – Brazil; 2 Brazil Instituto do Coração do Hospital das Clínicas da Faculdade de Medicina da Universidade de São Paulo - Departamento de Imagem Cardiovascular, São Paulo, SP – Brazil

**Keywords:** Chagas Disease, Tacchycardia, Ventricular, Magnetic Resonance Spectroscopy, Ablation Techniques/methods

## Introduction

The main endpoint of ventricular tachycardia (VT) ablation includes elimination of the abnormal substrate on remapping and rendering non-inducible VT with programmed ventricular stimulation. ^1 , 2^

Cardiac magnetic resonance (CMR) has been increasingly used as a tool for locating the arrhythmogenic substrate of patients with scar-related VT. The images can be integrated with the electroanatomical mapping to guide ablation to the identified channels responsible for the VT-reentry circuits, optimizing the ablation results. ^3^ However, until now, CMR has not been used to confirm the complete substrate elimination and effectiveness of VT ablation.

In this manuscript we report a case of a patient with Chagas disease cardiomyopathy ^4 , 5^ who underwent endocardial VT ablation, guided by VT mapping and substrate modification. We report, for the first time, the use of a post-procedure CMR for confirmation of VT-substrate elimination.

## Case Report

A 68-years-old male patient with Chagas disease presented with palpitations due to sustained monomorphic VT (Figure 1A). The VT was terminated with direct current cardioversion. The patient reported a prior episode of syncope and was receiving amiodarone 200mg due to non-sustained VT on Holter. The echocardiogram showed left ventricular ejection fraction (LVEF) of 40%, left ventricular (LV) dimensions 67x58 mm, lateral wall akinesis and inferior wall hypokinesis. The set of findings suggests that the patient was in stage B2 of the disease, according to the evolutionary classification of heart failure in Chagas. ^5^ An abdominal computed tomography (CT) scan was performed to evaluate for megacolon. A three-dimensional late gadolinium enhancement CMR (3D-LGE-CMR) was performed (Phillips ^®^ Achieva 1.5T system), using Navigator tool for respiratory compensation (3D-LGE sequence), and the raw files were then exported to ADAS ^®^ software (Galgo Medical, Barcelona, Spain) for imaging processing to evaluate scar distribution and presence of corridors. An inferior, lateral, basal, and apical scar with 19.4g (14.83% of LV mass) in the border zone and core and two corridors related to the inferior and basal scar were identified (Figure 1A). The corridor mass was 1.72g and both were on endocardial layers (10 and 30%), in which corridor #1 was in the lateral portion of the scar and #2 was related to the mitral annulus.

**Figure 1:** – A) Before ablation three-dimensional late gadolinium enhancement cardiac magnetic resonance processed in the ADAS ® software, showing a heterogeneous infero-basal scar with a core and a border zone, scar volume of 19.4g (14.83% of all left ventricular mass) and two corridors, corridor #1 close to the mitral annulus and Corridor #2 in the lateral aspect of the scar. B) same reconstruction but after the ablation; note the lateral extension of the scar, related to the area of ablation, with scar mass of 38.8g (26.6% of left ventricular mass) and no more corridors.

The patient was referred for VT ablation using CARTO 3 ^®^ (Biosense Webster/Johnson & Johnson, USA) system and recruited to a clinical trial, ^6^ in which was randomized to the “endocardial ablation only” group. The patient underwent endocardial voltage mapping showing inferior, lateral, and basal scar with fragmented potentials in the lateral portion of the scar and close to the coronary sinus. A hemodynamically stable clinical VT was induced (S1 600ms S4 310ms), with diastolic activity suggestive of a mitral isthmus VT, also correlated to corridor #2. Endocardial ablation (30W, total RF time: 24m4s) was performed with interruption of VT; the ablation was extended, eliminating all fragmented signals close to the annulus ( Figure 2 ). After remapping, abnormal potentials were still observed in the lateral part of the scar, in the region of corridor #1, so additional ablation was performed in that portion achieving complete substrate homogenization. A programmed ventricular stimulation (PVS) performed after ablation with S1 600ms and 430ms up to S4 did not induce any VT. Patient was discharged without an implantable cardioverter-defibrillator (ICD) and scheduled for re-evaluation in one month.

**Figure 2:** – Clinical ventricular tachycardia induced in the procedure, showing coronary sinus activation and CARTO ® activation mapping suggestive of a mitral isthmus circuit. Voltage mapping showed infero-basal scar, with abnormal fragmented potentials on the lateral aspect of the scar and close to the mitral annulus. There were three different VT morphologies induced during the procedure. The tridimensional-late gadolinium enhancement cardiac magnetic resonance (20% layer) and computed tomography anatomy were offline exported to CARTO ® after ADAS ® processing, showing that the radiofrequency lesion set was related to the heterogeneous scar and presence of corridors

One month later, a second 3D-LGE-CMR was performed using the same equipment prior used, now showing 38.8g (28.6% of LV mass) of scar. Importantly, no channels were detected within the scar (Figure 1B). During a second electrophysiological study using the same protocol of PVS, VT was still not inducible. Patient was discharged without ICD implantation, maintaining use of amiodarone 200mg. In a follow-up of 5 years, the patient was maintained under the same dose of amiodarone, with EF of 48% on echocardiogram, with no VT recurrence, and without ICD implantation.

## Discussion

The classical endpoint of VT ablation is non-inducibility of arrhythmias after the procedure, ^7 , 8^ but the complete elimination of all the abnormal electrograms ^9^ also correlates with a lower risk of VT recurrence. Although VT recurrence may occur due to disease progression, incomplete elimination of the arrhythmogenic substrate appears to be the main mechanism. ^10^ A *pre-ablation* CMR is commonly used to locate the corridors within the scar, a finding that is correlated with worse outcomes in chronic Chagas cardiomyopathy, and may guide VT ablation. ^5 , 11^ In this report we describe the use of repeated CMR after ablation to confirm that all such corridors have been eliminated.

Our patient had a hemodynamically tolerated VT, moderate LVEF (40%) depression, without extensive scar (19g) and only two corridors identified on CMR. During ablation, VT was mapped and interrupted without reinduction, and all abnormal potentials were eliminated. At the occasion, we decided not to implant an ICD, and to repeat a control CMR in one month followed by an electrophysiological study. Post-ablation CMR confirmed that all corridors were eliminated, together with a negative electrophysiological study, allowing for discharge of the patient without an ICD. ^12^ Five years later, the patient remained free of syncope or VT recurrence, without depression of LV function (48%), maintaining the use of a moderate dose of amiodarone (200mg), same he used before index VT episodes, and without an ICD.

The time necessary for the radiofrequency lesions to be identified on CMR is not completely known, since acute lesions can present edema and microvascular obstruction regions. In this case we repeated CMR – in the same machine and with same personnel –, and also the electrophysiological study one month after the index procedure, a timeframe that should show a more consolidated pattern of the lesion. ^13^

It is noteworthy that, since Chagas Disease is a progressive condition, with a natural history of progressive fibrosis, ^5 , 14^ new reentrant VT circuits may form, so the strategy of withholding the ICD still needs to be tested in a randomized clinical trial.

This research letter, however, supports an investigative application on the use of a control CMR to evaluate substrate modification after ablation, allowing patients with less extensive scar and without severe LV dysfunction to safely remain without ICD implantation.
